# Effects of control strategies on gait in robot-assisted post-stroke lower limb rehabilitation: a systematic review

**DOI:** 10.1186/s12984-022-01031-5

**Published:** 2022-06-03

**Authors:** Silvia Campagnini, Piergiuseppe Liuzzi, Andrea Mannini, Robert Riener, Maria Chiara Carrozza

**Affiliations:** 1 IRCCS Fondazione Don Carlo Gnocchi ONLUS, Via di Scandicci 269, 50143 Firenze, FI Italy; 2 Istituto di BioRobotica, Scuola Superiore Sant’Anna, Viale Rinaldo Piaggio 34, 56025 Pontedera, PI Italy; 3 ETH Zurich, Rämistrasse 101, 8092 CH Zürich, Switzerland; 4 Balgrist University Hospital, Forchstrasse 340, 8008 CH Zürich, Switzerland

**Keywords:** Robot-assisted rehabilitation, Control Law, Stroke, Neurorehabilitation, Lower limb, Gait Determinants

## Abstract

**Background:**

Stroke related motor function deficits affect patients' likelihood of returning to professional activities, limit their participation in society and functionality in daily living. Hence, robot-aided gait rehabilitation needs to be fruitful and effective from a motor learning perspective. For this reason, optimal human–robot interaction strategies are necessary to foster neuroplastic shaping during therapy. Therefore, we performed a systematic search on the effects of different control algorithms on quantitative objective gait parameters of post-acute stroke patients.

**Methods:**

We conducted a systematic search on four electronic databases using the Population Intervention Comparison and Outcome format. The heterogeneity of performance assessment, study designs and patients’ numerosity prevented the possibility to conduct a rigorous meta-analysis, thus, the results were presented through narrative synthesis.

**Results:**

A total of 31 studies (out of 1036) met the inclusion criteria, without applying any temporal constraints. No controller preference with respect to gait parameters improvements was found. However, preferred solutions were encountered in the implementation of force control strategies mostly on rigid devices in therapeutic scenarios. Conversely, soft devices, which were all position-controlled, were found to be more commonly used in assistive scenarios. The effect of different controllers on gait could not be evaluated since conspicuous heterogeneity was found for both performance metrics and study designs.

**Conclusions:**

Overall, due to the impossibility of performing a meta-analysis, this systematic review calls for an outcome standardisation in the evaluation of robot-aided gait rehabilitation. This could allow for the comparison of adaptive and human-dependent controllers with conventional ones, identifying the most suitable control strategies for specific pathologic gait patterns. This latter aspect could bolster individualized and personalized choices of control strategies during the therapeutic or assistive path.

## Introduction

In the context of the digital revolution, there is a new paradigm for which digitalisation is approached in a sustainable and accessible way. Data is seen as a resource with great potential for the improvement of social and economic problems, as well as the growth of productivity and innovation.

In this framework, robotics has an important role in collecting new patient-specific data and using it to provide support during therapy or daily life assistance, especially when leveraging exoskeletons with embedded Artificial Intelligence (AI) algorithms. Nowadays, AI algorithms are increasing the implementation efficacy of learning processes and are capable of collecting and labelling new data almost instantly. This, viewed through the iron triangle framework of healthcare systems, could bolster accessibility, improving quality while cutting costs [1, 2]. In healthcare, the application of such a new framework could lead to improvements in terms of personalised *therapies* or innovative treatments. Moreover, in an *assistive* context, user-tailored devices could promote their accessibility and distribution in daily life, fostering the long-term improvement of the quality of life of patients in chronic conditions.

### Stroke

There is already consistent evidence of the beneficial effects of robot-aided treatments of the lower limbs after stroke [3]. Such evidence is paving the way for commercial and research solutions that show positive effects on the recovery of patients during their acute or chronic post-stroke phase [4].

In an attempt to define *stroke*, in the 70 s, the World Health Organization gave the following definition: “neurological deficit of cerebrovascular cause that persists beyond 24 h or is interrupted by death within 24 h” [5]. Either due to a lack of blood flow (ischemic) or due to bleeding (haemorrhagic), a stroke can have serious consequences on the patient, making it the fifth cause of death and first for long-term disability [6, 7]. On this matter, neuroplastic shaping has been found to be fundamental for improving functional outcomes after a stroke [8, 9]. From the classical work of Wolpert et al., [10] it is known how learning through repetitions speeds up the formation of priors and how including rest periods and spacing rehabilitative sessions improves learning rates and reduces retentions rates [11, 12]. Neurologically, high-dose rehabilitation programs are most likely to induce permanent modifications in neural plasticity [13] and increase cortical excitability [14], even if the exact dose still must be defined according to the stage of the post-stroke recovery [15].

One of the pillar arguments in this field is the Cochrane review from Mehrholz et al. [3], which highlights, for all the previous reasons and many more, the importance of structured evidence for assessing the best conditions to provide the treatment. Indeed, among the key aspects for a beneficial recovery, there is the manner with which the rehabilitation treatment is delivered both in terms of intensity (duration, repetitions and frequency) and modalities [16].

### Control strategies

In the case of robot-assisted treatment it is crucial how the physical human–robot interaction is handled [2]; three factors concur to the motion of a combined dynamic system as the patient-exoskeleton: (1) the rigidity of the link (2) the mechanical response of both components and (3) the control laws of the active parts. The ***rigidity*** of the devices is inherently connected to the mechanical structure. Indeed, while soft materials intrinsically have varying compliance, rigid ones present a generally constant component of stiffness. On the other hand, the ***patients’ mechanical responses*** are supposed to vary during the treatment (improvement on gait phases, higher muscle force, etc.…) or in some cases, within the rehabilitation session (increase in fatigue levels, falls).

Henceforth, the only way to cope with a varying stiffness of the links and with always changing user motion intentions is a well-versatile and adaptive ***control strategy***.

The mechanical structure of robots is highly associated with these aspects, thus, it is important to differentiate between end-effector robots and exoskeletons. More specifically, in end-effector devices, movement is initiated through a unique distal contact point. Then, movement is indirectly transferred to all adjacent joints. Exoskeletons, on the other hand, wear the user and, after proper alignment of the rotation axis of the device and user’s joints, directly provide movement onto the joints.

Given the multiple contact points of the exoskeleton with the human, control strategy design for the physical human–robot interaction is inherently more challenging in the case of exoskeletons, rather than end-effector ones [17]. Also, end-effector robots are known to suffer from a scarce control of the proximal joints in the limb (located between end-effector connection and trunk), which could result in abnormal or even dangerous movement patterns. This results in an inherent advantage of exoskeletons, namely the presence of mechanical endstops concurring in containing joint hyperextension. For this reason and the inherently different underlying control problem, we focussed only on exoskeleton devices, both treadmill-based or leg-orthotics ones.

Strategies have been previously classified in position and force controllers. Position control drives the gait onto a fixed mode, forcing the user to follow a pre-defined or adaptive trajectory, usually with rather low compliance. On the other hand, force control relies on a force signal produced by the limb contraction and its interaction with the mechanical parts of the device. Force or torque sensing devices have high determinacy, making the force control of exoskeleton devices steady and reliable. Conversely, these sensors often require rigid mechanical structures to produce an accurate force estimate, which makes this strategy not very common in modern soft exoskeletons. Position controllers, on the other hand, are strongly influenced by small errors on relative position variations, which may yield significant contact forces, if the interaction stiffness is not too low. Therefore, by adding the knowledge of these forces, the robot task space could be split into two subspaces, as in the Lokomat [18, 19], achieving a higher cooperative robot behaviour with a hybrid force-position control. In the family of force control strategies, we must include impedance control. It aims to control the rigidity and damping between the device and the user, avoiding excessive forces at the interface. Furthermore, specific types of controller modalities have to be highlighted. A bang-bang controller is a feedback controller switching between two different states (also called on–off controller). Assist-as-needed (AAN) on the other hand, provides the minimal amount of robotic assistance required to fulfil the movement trajectory. The latter results in a commonly used strategy, maximizing the effort made by the patient, promoting his/her active participation. Lastly, tunnel or path control allows freedom of movement within a virtual tunnel of adjustable size around the predefined joint trajectory. The latter differs from pure position controllers by enforcing an error margin around the trajectory, increasing safety levels and compliance of the device mechanical response.

In addition, to enhance participation, augment human–robot interaction and promote adaptive neuroplasticity shaping, strategies based on biological signals have been developed (surface electromyogram, sEMG and electroencephalogram, EEG) [20]. sEMG is used to record the surface component of activity produced by the skeletal muscle [21]. It gives a non-invasive measure of human motor activity and, in opposition with the force sensing, it provides information about specific muscle groups' activity and not about the combination of all muscle groups. Furthermore, sEMG, or more in general EMG signals, allow investigating active motion intentions and synergies by evaluating activation timing and intensity of connected muscle groups. For what concerns stroke, EEG-based prosthetic control was not found in an extensive number of applications, due to the high likelihood of a lesion being in the brain motor function area, making it unable to produce regular EEG signals.

Currently, several review papers are available either on an effective comparison between robot-assisted treatment and conventional therapy or the screening of the most used control strategies. However, up to our knowledge, none of them is addressing the association between controllers technical requirements and their expected outcome.

For this reason, we systematically reviewed the control strategies currently used in lower-limb rehabilitation robots for stroke patients, providing a classification of the control strategies and the outcome measures adopted. A comparison of control techniques and mechanical requirements needed in *assistive* and *therapeutic* environments is performed. Furthermore, we investigated whether a preferred association exists between the different solutions designs and the outcome measure used to assess treatment benefit. The remaining of the paper is organized as follows. Section 2 describes the methods used for the review. Section 3 reports the results on the included papers, while in Section 4 we discuss the results and the limitations of the study. In Section 5, a brief conclusion is given and future outlooks are summarised.

## Methods

A systematic review was performed following the Preferred Reporting Items for Systematic Reviews and Meta-Analyses (PRISMA) guidelines [22].

### Selection criteria

The selection of the study was performed using the Population, Intervention, Comparison and Outcome (PICO) framework [23].

For what concerns the type of study, we selected all types of primary studies, excluding overviews and reviews.

As participants, we selected adults (age greater than 18 years old), post-stroke patients in the chronic or sub-acute phase (time from event greater than 1 month). Papers considering mixed populations (with different aetiologies than stroke or mixed among post-stroke and healthy) were excluded unless results could be retrieved for the post-stroke subjects only.

For what concerns the intervention, we focussed on lower limb exoskeletons for both *therapeutic* and *assistance* purposes. More specifically, we selected papers addressing the control strategy used for the physical human–robot interaction and performing experiments on the post-stroke patients. Papers investigating single control strategies or comparing different ones were included.

Finally, regarding the outcome, we selected papers investigating variables related to objective and quantitative gait parameters, thus, excluding papers evaluating exclusively non-quantitative clinical scales or empirical evaluations. From papers containing both quantitative and qualitative parameters, only quantitative ones were retained for further consideration.

### Search method for identification of studies

A systematic search was conducted in the following databases: PubMed, Web of Science, Scopus and CENTRAL. The string search was built using the PICO format [23], using as main keywords the terms: “Stroke”, “Robot”, “Exoskeleton device”, “Lower extremity” and “Control”.

Once the results were extracted, two independent reviewers (SC and PL) performed the screening on title and abstract first and full text at last. A third reviewer was involved in case of disagreements (AM). During this phase, only papers in English were considered eligible for screening. The selection concerning outcomes was not applied during the search phase; it was involved in the screening phase only.

### Data collection

For the data collection, the CHecklist for critical Appraisal and data extraction for systematic Reviews of prediction Modelling Studies (CHARMS) was used [24]. The data extracted from the included studies concerned:

- Source of data
- Participant characteristics (age, number, specifications of the stroke event)
- Setting (monocentric or multicentric, therapeutic or assistance setting)
- Study design (Randomised Controlled Trials (RCTs), Controlled Trials (CTs) or none of the previous NCTs)
- Description of the device (actuated joints, actuation type, structure characteristics, Degrees of Freedom (DoFs))
- Control strategy used (type, presence and type of control input)
- Outcomes (measures used and timing)

### Data synthesis

Results were displayed through narrative data synthesis since a meta-analysis was excluded due to the heterogeneity of study designs.

Firstly, a description of the population and devices characteristics was provided. Then, results about the control type, the input provided to the closed-loop control strategies and the evaluated outcome were displayed both through a general description and a subgroup division based on soft or rigid robotic structure. A further distinction of the results between *assistive* and *therapeutic* intended purpose of the devices was presented. Such distinction between the two groups was given according to the indications on the device provided by the authors and either confirmed or changed given the specific experiments conducted within the selected paper. More specifically, papers analysing the evolution of the outcome in a longitudinal way, aiming at detecting an improvement of the patient after the robotic rehabilitation sessions were considered as *therapeutic*. On the other hand, studies comparing the outcome of the patients performing a task with and without the device, within a specific evaluation time window, were considered as *assistive*. As previously mentioned, in case of disagreement among the generic indication of the authors, according to the nature of the device, and the proposed results, the distinction was driven by the type of analyses conducted.

To conclude, a summary of all the parts treated singularly was provided in order to give a comprehensive view of the control strategies used on the different devices and the outcome selected.

## Results

**Fig. 1:**
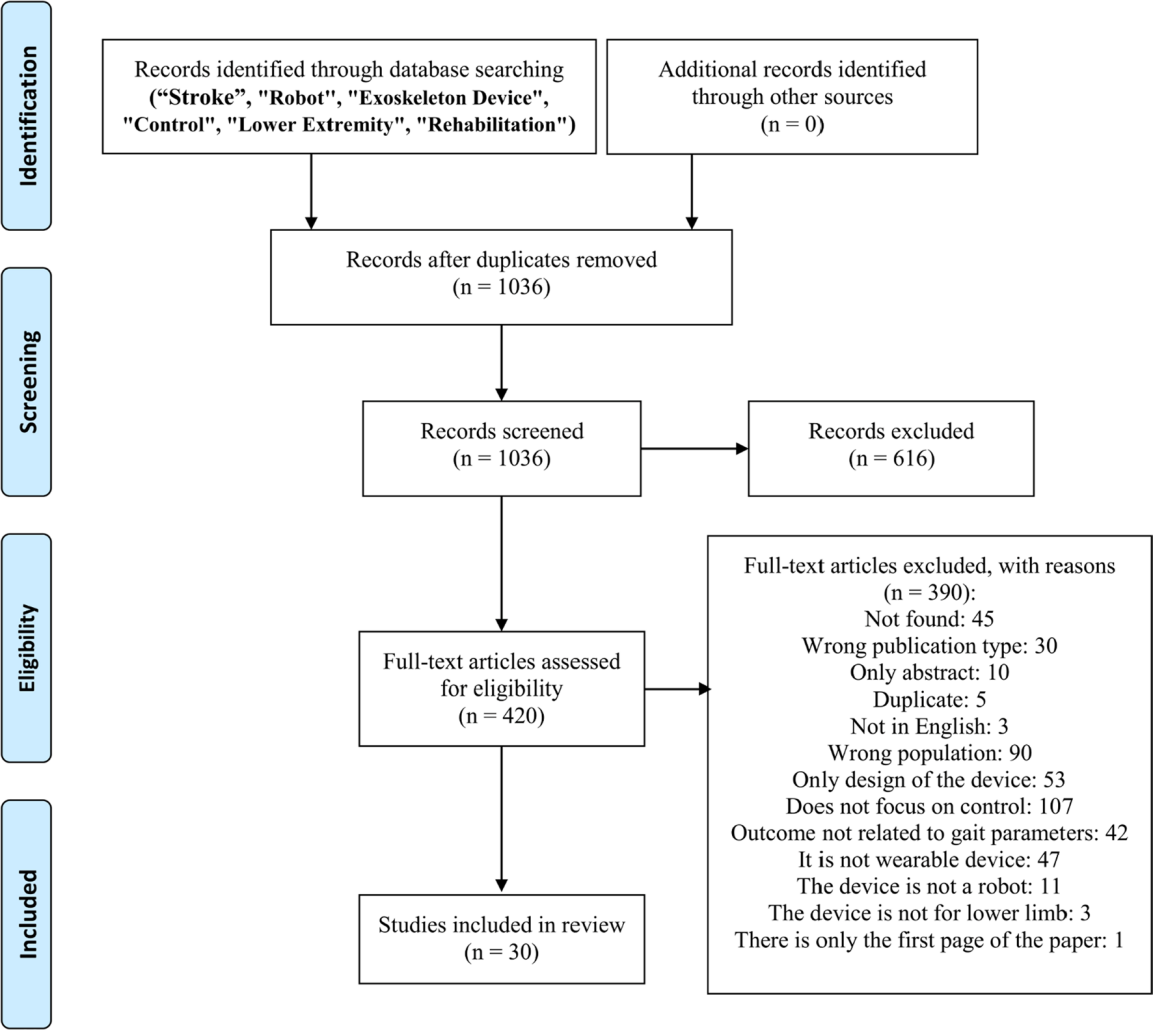
PRISMA workflow diagram

In this study, a total number of 30 papers was included out of 1036. The two main reasons for exclusion were related to the absence of experiments on post-stroke patients or the absence of a detailed control strategy description of the device (Fig. 1). Forty-two papers were excluded since they did not have any quantitative gait parameter among the performance metrics.

With no constraint on the string search temporal span, the papers included were dated from 2009 up to 2020, with a publishing median year dated in 2017. In the studies included, 258 participants were enrolled, 11 of which were healthy controls and 247 post-stroke patients. Enrolled cohorts ranged from 1 to 50 patients per study.

For what concerns the study design, only three were RCTs [25–27] and one CT [28], corresponding to the four papers with the higher numbers of patients included. Of these papers, only Villa-Parra et al. [28] distinguished experimental and control groups among post-stroke patients and healthy subjects, whilst in the remaining three [25]–[27] the two groups were distinguished on the treatment type. Thus, among the 247 post-stroke participants, 194 actually performed robotic treatment.

The 247 post-stroke participants had a mean age of 52.9 (std 8.0) years old, given that 3 papers did not report this information [29–31] and 1 paper broadly reported the inclusion of participants over 18 years old [25]. Further details are reported in Table 1.

**Table 1:** Descriptive statistics of study settings, therapy intensity and exoskeletons characteristics

Article (Year)	CT (# controls)	Patients #			Age (yrs.)	Stroke phase		Therap or Assist	Training		Robot characteristics		Act. joints			Act type	Control type		Control inputs types (JK, JF, GRF, EMG)
		ML	BL	N/R		SA/CHR	Timing after stroke		Dose	Frequency	Soft/Rigid (Kg)	Active DoF	H	K	A		P/F/H	BB/AAN/T/I	
Awad (2017) [45]	N/A	11			49 ± 4	CHR	> 6 mo	A	N/A	N/A	S (4.09)	1			x	BC	P	N/A	JK-GRF
Quintana (2020) [34]	N/A	4			18–75	N/R	N/R	A	N/A	N/A	R (2.8)	1		x		JT	P—F.^a^	AAN	N/A
Forrester (2011) [46]	N/A	8			18–85	CHR	> 6 mo	T	30–60 min	18 in 6 w	R	1			x	C	F	AAN, I	N/A
Forrester (2014) [25]	18 Str	18			> 18	SA	< 50 days	T	30–60 min	~ 10	R	1			x	C	F	AAN, I	N/A
Forrester (2016) [47]	N/A	26			59 ± 4	CHR	> 6 mo	T	30–60 min	18 in 6 w	R	1			x	C	F	AAN, I	N/A
S. Hirano (2017) [36]	N/A	1			70	SA	= 17 days	T	40 min	20 in 4 w	R (5.7)	1		x		JT	F	N/A	JK-GRF
Van Asseldonk (2009) [29]	N/A	4			N/R	CHR	N/R	T	45 min	18 in 6 w	R	8	x	x	x	BC	F	I	JK
Murray (2015) [48]	N/A	1			39	CHR	> 3 mo	A	N/A	N/A	R (12)	4	x	x		JT	F	I	GRF
Murray (2014) [49]	N/A	3			39,42,69	CHR	> 3 mo	A	N/A	N/A	R (12)	4	x	x		JT	F	N/A	GRF
Banala (2009) [33]	N/A			2	N/R		N/R	T	3 h	15 in 3 w	R	2	x	x		JT	F	T	JK-JF
Bae (2015) [50]	N/A	3			29–63	CHR	N/R	A	N/A	N/A	S (0.3, textiles)	2			x	BC	P	BB	JK
Buesing (2015) [26]	25 Str	25			18–85	CHR	~ = 12 mo	T	45 min	18 in 6 w	R (2.8)	1	x			JT	F	I	N/A
Durandau (2019) [42]	N/A	2			37–72	CHR	N/R	A	N/A	N/A	R	3	x	x	x	JT	F	N/A	JK-EMG
Jia-Fan (2010) [35]	N/A	1	2		31–59		N/R	T	N/R	N/R	R (30)	2	x	x		JT	F	T, I	JK
Bishop (2017) [38]	N/A	2			39–53	CHR	~ = 16, 3 yrs	T	40 min	5 sessions	R	N/R	x			C	F	BB	JK-JF-GRF
Krishnan (2013) [40]	N/A	1			62	CHR	N/R	T	45- 60 min	12 in 4 w	R	2	x	x		JT	F	T	N/A
Krishnan (2012) [41]	N/A	1			52	CHR	N/R	T	90 min	12 in 4 w	R	2	x	x		JT	F	T	JK
Kwon (2019) [51]	N/A	1			49	CHR	> 10 mo	A	N/A	N/A	S (1.54)	1			x	BC	P	N/A	JK-GRF
Lee (2019) [27]	14 Str	14			62 ± 8	CHR	> 3 mo	T	45 min	12 in 4 w	S (2.8)	1	x			JT	P	AAN	JK
Martinez (2018) [39]	N/A	1			37	CHR	~ = 6 yrs	A	N/A	N/A	R (12)	4	x	x		JT	P	T-BB	JK
McCain (2019) [52]	N/A	6			50 ± 8	CHR	> 6 mo	A	N/A	N/A	R	1			x	BC	F	AAN	JK-GRF-EMG
Mizukami (2018) [32]	N/A	15			59 ± 15	SA/CHR	~ = 63–1383 days	A	N/A	N/A	R (5.8)	4	x	x		JT	F	I	JK
Villa-Parra (2020) [28]	11 Heal	3			53—58	CHR	~ = 9–21 mo	A	N/A	N/A	R (3.4)	1		x		JT	F	I	JK-JF-GRF
Roy (2018) [53]	N/A	14			18–85	CHR	> 6 mo	T	60 min	18 in 6 w	R (3.6)	1			x	JT	P	N/A	GRF
Sacco (2018) [30]	N/A	1			N/R	CHR	> 10 mo	A	N/A	N/A	R	6	x	x	x	JT	P	T	JK-JF
Swift (2010) [31]	N/A	3			N/R	CHR	> 12 mo	A	30 min	1 session	R (39)	4	x	x		HYD	H	N/A	JK-GRF
Van Asseldonk (2009b) [54]	N/A	5			57 ± 4	CHR	> 6 mo	A	N/A	N/A	R	8	x	x	x	BC	F	I	JK
Tanaka (2019) [44]	N/A	11			21–81	CHR	N/R	T	60 min	6–15 in 3 w	R	2	x	x		JT	F	N/A	JK-EMG
Yeung (2017) [37]	N/A	3			58,58,72	CHR	> 24 mo	A	N/A	N/A	R (1)	1			x	JT	F	N/A	JK-JF
Zadravec (2017) [43]	N/A	1			64	CHR	> 10 mo	T	15 min	31 in 10 w	R	3	x			JT	F	I	GRF

*CT* Controlled trial, *ML/BL* monoliteral/bilateral, *SA/CHR* sub-acute/chronic; H/K/A: hip/knee/ankle, *BC* Bowden cables, *C* cables, *JT* joint torques, *HYD* hydraulic, *P/F/H* position/force/hybrid, *BB/AAN/T/I* bang-bang/assist-as-needed/tunnel/impedance, *JK* joint kinematics, *JF* joint forces, *GRF* ground reaction forces, *EMG* electromyography, *N/A* information not applicable, *N/R* information not reported

For what concerns the time from the event, 24 papers focussed on chronic patients, 1 considered both chronic and sub-acute patients [32], 3 papers did not report this information [33–35] and the remaining 2 specifically focussed on sub-acute phase [25, 36]. Specifically, Hirano et al. [36] and Forrester et al. [25] included patients at 17 days and less than 50 days from stroke respectively. Diversely, 3 of the papers focussing on chronic patients included long-term event participants, with time from event greater than 2 years [37], ranging between 3 and 16 years [38] and at 6 years [39]. Regarding stroke aetiology, only two papers specifically declared among inclusion criteria the selection of ischemic stroke only [40, 41].

Regarding the experimental setting, 12 papers performed the experiments with an *assistive* purpose and presented the device for assistive applications. On the other hand, 15 papers performed experiments with a therapeutic aim, presenting the results as a comparison of the patients’ conditions before and after the treatment and proposing the device itself as a *therapeutic* device. Durandau et al. [42] did not specifically declare the purpose of the device, however, results were presented comparing different control modes. Instead, one paper presented the device as a *therapeutic* robot but performed the experiments comparing the patients’ outcomes on the same tasks with and without the device worn [31]. Thus, both papers were listed among the *assistive* ones. Finally, Jia-fan et al. [35] described a device aiming at *therapeutic* applications but reported the results in a descriptive manner, whilst in the paper from Zadravec et al. [43] the ultimate purpose of the device is not clearly stated, however, both the mechanical design and the results shown indicate for a *therapeutic* application. Hence, both papers were allocated to the *therapeutic* class.

**Fig. 2:**
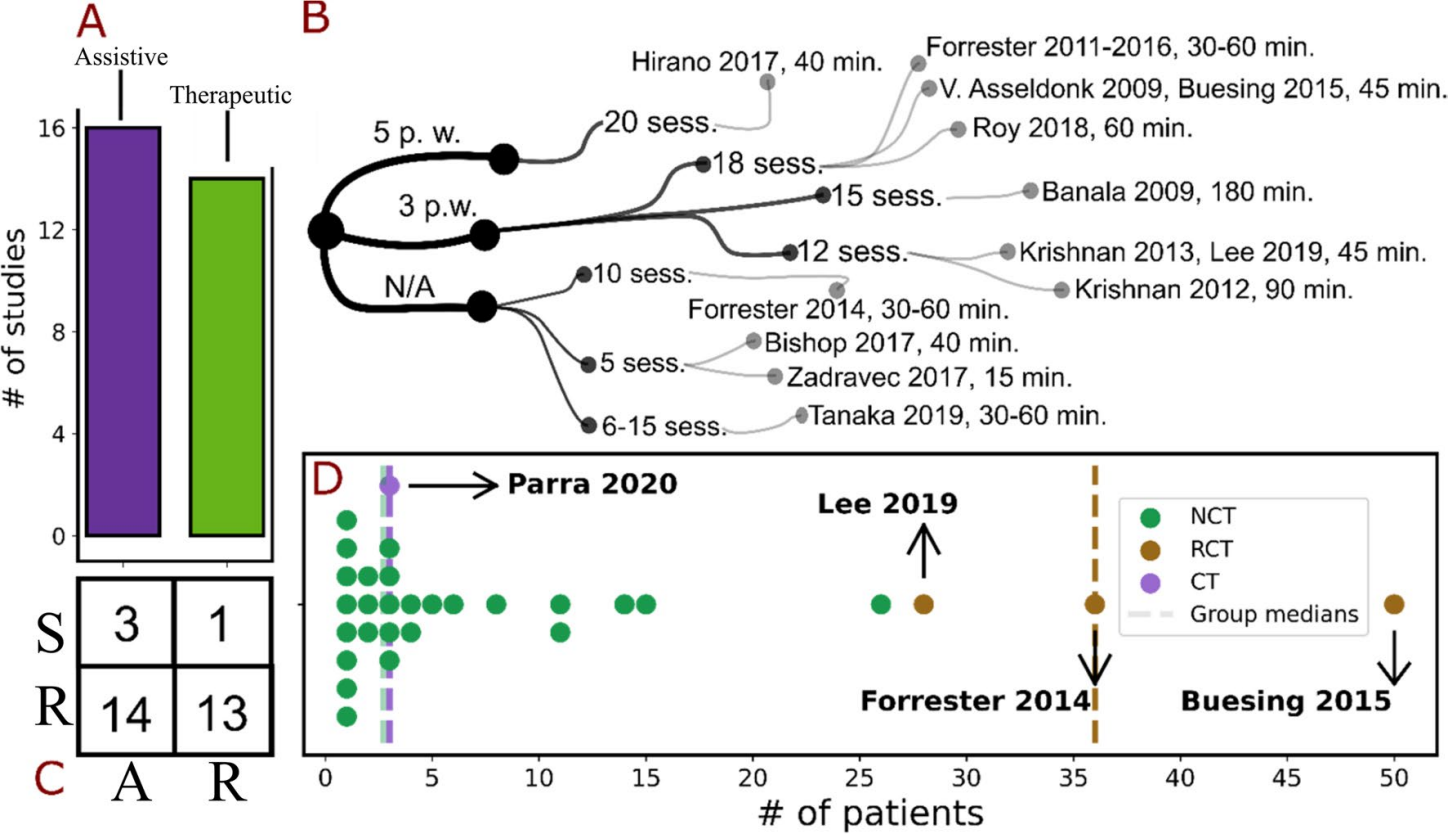
Description of study settings. Intended study destination (**A**), participants numerosity grouped for study design type (panel **D**) and exoskeleton configuration (R: rigid, S: soft) (**C**). In **B**, rehabilitation intensity is shown. Specifically, the first subdivision is done following the number of sessions per week (p. w.), the second according to the absolute number of sessions provided to the patients and the third indicates the duration of the sessions of each paper

Except for Jia-fan et al. [35], all the studies associated with the *therapeutic* setting reported information about the dose, frequency and duration of the intervention. Most of the papers selected a session duration of 45 – 60 min, except for Banala et al. [33], Krishnan et al. [41], and Zadravec et al. [43] performing 3 h, 90 min and 15 min sessions, respectively (Fig. 2).

The total number of sessions ranged between 5 [38, 43] and 31 [43], with an average of 13 sessions. Lastly, the preferred frequency selected was three sessions per week.

Regarding the robotic devices used, a first classification from the mechanical point of view distinguished them among *soft* and *rigid* devices. *Rigid* devices were used in most papers (26 out of 30), whilst *soft* devices were employed in 4 studies only. Among the device descriptions, the weight of all *soft* robots and only 12 of the *rigid* ones were reported. As reported in the contingency tables, most *soft* devices were used and tested for *assistive* scenarios, whilst only one was intended for *therapeutic* purposes [27]. The actuation of the devices was distinguished among hydraulic and electric, and among the latter, a further distinction was made based on the presence/absence of cables or Bowden cable systems. In detail, only one paper reported the use of a hydraulic actuated robot [31], with a weight of 39 kg, used for *therapeutic* applications. Among the electric actuation instead, 6 studies reported the use of Bowden cables, 4 studies reported the use of cables and the remaining 19 involved robotic devices with the direct application of a torque on the desired joint.

Moreover, 16 studies preferred a direct actuation of 1 joint only, 10 studies on 2 joints and 4 studies directly actuated all hip, knee, and ankle. Among the devices offering direct assistance on one joint only, four devices involved the hip, aiding only in the sagittal plane in two cases [26, 27] and both in the frontal and sagittal plane in the other two devices working toward the restoration of balance and gait symmetry [38, 43]. Villa-Parra et al. [28], Quintana et al. [34] and Hirano et al. [36] provided assistance on the knee only, whilst the remaining nine studies focussed on the ankle.

**Fig. 3:**
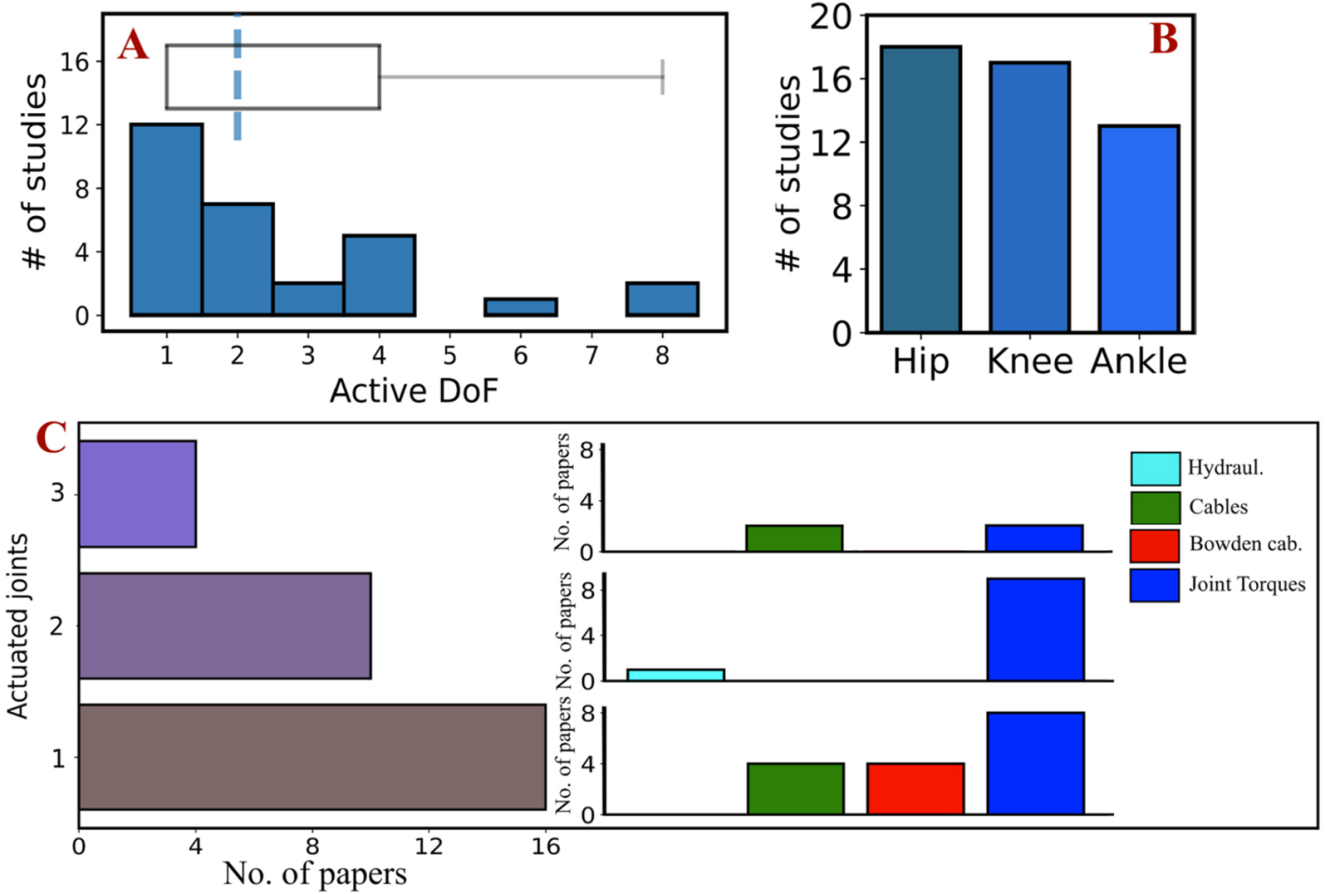
Description of exoskeleton mechanical properties. Exoskeletons active DoF (**A**) and studies distributions of actuated joints (**B**). Number of papers with respect to the number of actuated joints **C** with a subdivision for each group (one, two, three) of the actuation method used

It was noticeable how cables or Bowden cables solutions were preferred for devices with direct actuation of the ankle only, or equally preferred as direct torques of electric motors in those where the three lower limb joints were involved (Fig. 3). Contrarily, devices with two joint actuation and with the only actuation of the knee or hip preferred direct torque. An exception of these findings is represented by the study from Swift et al. [31], the only study reporting about hydraulic actuation, and the study by Bishop et al. [38], for which cables were used in a pelvic device for balance recovery. In addition, 15 studies performed experiments overground, 11 on the treadmill, 2 studies both with treadmill and overground. Moreover, three studies performed experiments using body-weight-support without specifying the percentage of weight supported [35, 43, 44].

Regarding the control strategies implemented, studies were first broadly distinguished among position (8 studies), force (22 studies) and hybrid (1 study) control types (not mutually exclusive, as Quintana et al. [34], who adopted both force and position control strategies for comparison). Going more in detail, controls were further described through the non-exclusive classes of bang-bang (3 studies), tunnel (6 studies) and assist-as-needed (6 studies) types. Among papers adopting force-control strategies, 11 (50%) of them directly controlled the impedance/admittance of the device.

**Fig. 4:**
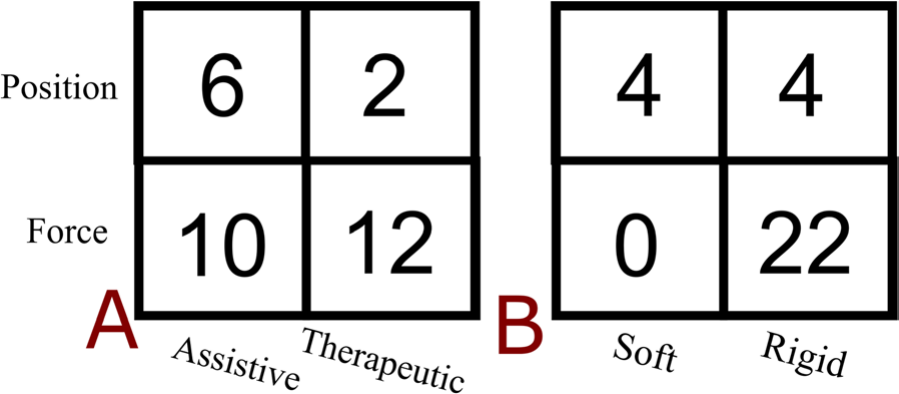
Control strategies design preferences. 2 × 2 contingency tables of controller type distributions compared with the study setting (**A**) and the exoskeleton rigidity (**B**)﻿

In the following contingency tables (Fig. 4), the relative frequencies of *assistive/therapeutic* and rigid/soft devices were presented on the subgroups generated by the control types. It is visible how studies with position control are predominantly associated with *assistive* applications, whilst on the contrary, force control types are for the majority related to *therapeutic* ones. Similarly, a trend in the use of control types was found depending on the mechanical structure of the robots. In particular, in all soft robots, position control was used, while the majority of rigid robots were associated to force control strategies.

For what concerns the control input signals used, most studies (n = 20) used joint kinematics variables (e.g. joint angles, velocities, gait phases, etc.) while 5 studies took as input the forces at the joints. Interestingly, these five studies also included information on joint kinematics. Additionally, Ground Reaction Force (GRF) and EMG signals were used in 11 and 3 articles, respectively. Only Bishop et al. [38] and Villa-Parra et al. [28] developed control strategies using joint kinematics, forces and ground reaction forces together.

Concerning the outcomes, we included in this review only quantitative and objective gait parameters. Hence, papers including exclusively subjective evaluation of the clinical professionals and qualitative scales were excluded. On the other hand, from those containing both quantitative and qualitative parameters, results were extracted for solely the quantitative ones.

According to the measurement used, the resulting outcomes were classified into:

- *Kinematic gait parameters*: this class of parameters includes those variables representing the gait kinematics except the ones used as symmetry markers. The majority of the studies reported results in this category (24 studies).
- *Dynamics and synergies*: this category includes the outcomes related to muscle activity or gait dynamics. A total of 15 studies included these outcomes. However, only three studies [27, 28, 42] used outcome measures derived from EMG signals.
- *Symmetry*: this class of outcomes was considered in 12 studies, and it represents any metrics used for evaluating the symmetry of the gait.
- *Cost of walking*: this category is related to any measurement related to fatigue or metabolic cost. Only three studies investigated these types of outcomes.
- *Objective quantitative scales:* this class groups outcomes obtained through clinical tests such as Time Up and Go or 6 Minutes Walking Test, for which a final objective assessment is provided. A total of six studies used outcomes belonging to this category.
- *Ability to match a reference template:* this class of outcomes is the most heterogeneous, involving self-selected parameters to evaluate the ability of the control strategy to match a reference template provided or, more generically, to optimally behave during the experiments. Five studies used these outcomes.

Specifically, the most common outcome measures addressed among the included studies were mean speed (13 studies), stride length (7 studies), ankle ROM (6 studies), step length and step length symmetry, with 5 studies each. Overall, 22 studies considered outcomes belonging to more than one category. On the contrary, among those considering multiple outcomes, Lee et al. [27] and McCain et al. [52] considered outcomes belonging to four different categories.

The specific outcomes with respect to the previously described classes are presented in Table 2. After analysing the outcome as a standalone characteristic, we investigated differences between subgroups given by force/position control as well as therapeutic/assistive devices. No particular trend was found in the choice of the outcome for each control type or vice-versa. Similarly, the intended use of the device (assistance vs. therapeutic) does not affect the evaluated outcomes. Overall, the distribution of the outcomes within the individual subgroups respects the distribution of the overall outcome. The only outcome class ruling out is the *Ability to match a reference template* which has greater distribution in studies adopting force controllers (five out of six studies).

**Table 2:** Study outcomes grouped in functional categories

Kinematic Gait Parameters	Dynamics & Synergies	Symmetry	Cost of Walking	Objective Quantitative Scales	Ability to match a reference template
Ankle ROM [30, 33, 37, 45, 51, 54]	GRF x [43, 52, 53]	Gait time symm. [44]	Oxygen consumption [45]	10MWT [34, 44]	Exo-applied torque [52]
Knee ROM [30, 31, 33]	GRF y [43]	GRF z symm. [38, 41]	Energy consumed per meter [52]	6MWT [40, 41]	Exo-applied power [52]
Hip ROM [30]	GRF z [38, 41, 45, 52]	Interlimb Propulsion symm. [45]	Net metabolic energy cost [27]	Human Autonomy Index [53]	Avg. % change in joint angles [32]
Cadence [26, 30, 32, 46]	AP impulse [47]	Joint Angles symm. [32]		TUG [40, 41]	Control parameter [33]
CoP length [43, 47]	Index of norm. variability [42]	Spatial symm. [38, 50]			Target tracking % error [41]
Mean speed [25]–[27, 29, 32, 33, 36, 40, 41, 46]–[49]	Joint torques [35]	Temporal symm. [26, 27]			Tracking error [42]
Covered Distance [40]	Muscular frequency [28]	Stance time symm. [38, 44, 50]			Knee-Hip 2D trajectory [39]
Foot trajectory [50]	MVC [27]	Step time symm. [25, 50]			
Forward tilting angle [37]	Paretic propulsion [50, 51]	Step length symm. [25, 48]–[51]			
Gait cycle time [44]	MAV EMG [42]	Stride length symm. [38]			
Heel-first foot strikes [53]	Pelvic interaction forces [43]	Stride time symm. [50]			
Leg angular velocity [37]		Stride velocity symm. [38]			
Paretic swing angle [53]		Swing time symm. [38, 51]			
Trailing limb angle [52]		sEMG RMS mean diff. [28]			
T single/double supp. [26, 44]					
T_paretic_ single/double supp. [46, 47]					
Single leg balance [41]					
Step time [26, 43]					
Stance time [26, 38]					
Step height [29, 54]					
Step length [25, 26, 29, 32, 43]					
Step width [43]					
Stride length [26, 27, 31, 38, 46, 48, 49]					
Stride velocity [38]					
Stride time [31]					
Swing time [26, 29, 38]					

*ROM* range of motion, *CoP* centre of pressure, *GRF* ground reaction force; AP: anteroposterior, *MAV* mean absolute value, *MVC* maximal voluntary contraction, *10MWT* 10 m walk test; *6MWT* 6 min walk test, *TUG* time-up and go

## Discussion

In post-stroke therapy, the physical interaction between the physiotherapist and the patient is one of the key aspects to be considered for the optimisation of learning processes and neuroplasticity shaping during motor rehabilitation. When looking at rehabilitation robotics, specifically with exoskeletons, this latter concept inherently translates to human–robot interaction. Control strategies are crucial in defining how the interaction is handled. The control law continuously responds by balancing the necessary assistance and need for the patients’ maximal participation. This task, to be carried on in daily rehabilitation scenarios, requires fine sensing capabilities, robust safety measures and the ability to impress forces coherently with the task performed [55, 56].

The best way to translate such a technical aspect into the clinical application, and to fully understand the impact on the patient-robot interaction, is to understand from early steps the preferred type of control strategy for robot type and the most common outcomes associated with each control strategy.

For these reasons, we performed a systematic review on control strategies currently used in *therapeutic* and *assistive* exoskeletons for stroke patients, studying both the control characteristics and the outcomes of the experiments. In order to investigate this very specific aspect, we selected papers describing an early stage of experiments, aiming at an assessment and tuning of the robot control.

Indeed, the two main reasons for exclusion were related to the absence of experiments on post-stroke patients or the absence of technical description of the device control strategy. These two criteria were often mutually exclusive since they are related to two different phases on the device Technology Readiness Level (TRL) milestones (i.e. of development and clinical validation of the device). Confirming this, the papers included presented limited sample sizes and rarely, except for Villa-Parra et al. [28], Lee et al. [27], Forrester et al. [25], and Buesing et al. [26], Controlled Trails designs were encountered.

For what concerns the experimental setting, we did not constrain the selection, opening both to *therapeutic* and *assistive* purposes. Among the papers implementing a *therapeutic* study design, hence a study with multiple sessions per patient and a “pre-post” training comparison, a frequency of at least three sessions per week was the most diffuse, coherently with previous findings [57–59] and clinical practice. At the same time, four articles did not report the frequency of the treatment but only the absolute number of sessions, which in our opinion, is not enough to define the training intensity (Table 1). However, defining optimal training levels is not the focus of the review, thus, we did not investigate this aspect.

In line with these considerations, we also included papers with an *assistive* study design, hence where the experiments took place in a “with/without” exoskeleton condition, without any form of continuous repetitive training.

However, the classification among *therapeutic* or *assistive* studies is not only based on the experimental setting and presentation of the results, but also the objective application of the device. In some cases, as in Swift et al. [31], it was declared a therapeutic intent whilst pre-post comparisons were proposed as the results, with only one session done with the device. Moreover, in cases like Jia-fan et al. [35], results were presented in a descriptive way, thus, the classification as the *therapeutic* device was provided on the intended application of the device exclusively.

The *therapeutic* and *assistive* scenarios inherently differ for what concerns hardware and software requirements and for the safety levels needed [2, 60]. Indeed, we noticed how—with respect to hardware -, the *assistive* scenario called for lighter and less bulky devices, thus, for solutions with soft links between fixed points of the joints (Fig. 2C).

Maximal neuroplastic shaping is obtained if the assistance decreases when the patient muscular strength and force increase [61]. For this reason, if during the rehabilitation the patients’ gait is robustly driven within a predefined trajectory/path, and forces are provided independently on the patients’ remaining motor capability, the existing muscular force is not stimulated to emerge and strengthen. In our work, this concept was supported by the results that most of the position-controlled exoskeletons were used for assistive purposes (6 out of 8). The latter findings allow us to further speculate on how more precise trajectory-following behaviour is required in later steps of the motor recovery process of stroke patients and in non-structured daily environments, where assistance needs to be provided. However, these considerations are still an open aspect, as in the literature the choice of the best performing control strategy, in terms of neuroplastic shaping, is still controversial [62]. Lastly, all *assistive* studies focussed on patients throughout the chronic phase after stroke, instead than in the sub-acute period. On the other hand, 12 out of 14 *therapeutic* studies implemented a force controller, suggesting how the course of sub-acute recovery might require different phases in the support, hence necessitating for adaptive control, especially on the early phases and decreasing support levels in the long term. Coherently, when evaluating how control strategies vary between soft and rigid devices, we noticed that all the soft exoskeletons included adopted a position controller with joint kinematics inputs. The force sensors requirements to have a rigid sensing structure may explain the force controllers preference of operating with rigid structures instead of a calculation of dispersion forces in the soft strain.

We defined as control input the inputs to the controller both in the feedforward or in the feedback direction, including all signals used within the control strategy, independently on the type of sensors used. Signals were included, without conditioning on the targeted controller subtasks (e.g. gait segmentation, input to proportional-integrative-derivative (PID) controllers or neural networks, evaluation of assistance coefficients, etc.…), as long as they contributed to the control of the device. We grouped the signals in joint kinematics, joint forces, EMG signals and GRF. Since our focus was on the control strategies implemented, we decided to focus only on those signals in input to the control unit, neglecting any kind of feedback provided to the participants, such as auditory feedback or visual feedback.

It was noticed that all the articles used at least joint kinematics or ground reaction force to conduce gait. Moreover, no controllers implementing EMG and joint forces as their sole feedback input were found. In all cases, joint kinematics or ground reaction forces appeared necessary to provide either a fine segmentation of the gait phases or a continuous adaptation of the control laws. Moreover, no position controller is used as input the EMG signal, as the dynamic content contained in EMG signals is not helpful for this type of control [63].

Concerning the outcomes assessed during the experiments, in this review only quantitative objective gait parameters were considered. Indeed, we did not consider subjective evaluations, subjective clinical scales and self-compiled questionnaires since these methods do not offer a systematic evaluation of performances and are subjected to high inter- and intra-rater variability. Classical biomechanical determinants, such as mean speed or step length, were addressed by the most conspicuous group of studies (13 out of 31). However, as authors moved away from evaluating a classical biomechanical parameter toward higher-level gait functioning, a smaller consensus was found in measures selection and reporting.

For this reason, we grouped outcomes according to the type of represented measure, namely in kinematic gait parameters, dynamics and synergies, symmetry, cost of walking and objective quantitative scales.

Reasonably, the sensors used to provide inputs to the controller were the ones used to assess outcomes. Specifically, all the papers evaluating kinematic gait parameters utilised as controller input at least data taken from joint kinematics. Interestingly, within the *muscular dynamics and synergies* outcome group, only Durandau et al. [42] also used the EMG signal to control the device.

Measures of cost of walking and symmetry indexes were found to be highly heterogeneous among authors. No specific trend was noted on the choice of the outcome conditioned either to an *assistive* or *rehabilitation* experimental set-up or to a position or force control type. Coherently with expectations, most studies investigating outcomes related to *Ability to match a reference template* adopted a force controller. Indeed, in position controllers, the error with respect to a reference template is directly involved in the control laws. On the contrary, in often highly-compliant dynamics needed by force controllers, such parameters may retain a greater informative content. Despite grouping remarked how the most common evaluation metrics are related to kinematic gait parameters (e.g. speed, ROM and step length), no relevant trend was encountered between outcome groups and either control strategy or devices intended use.

Known limitations of the study are related to the methodology of the search. First, it was conducted selecting only results in English (three papers were excluded for this reason). Moreover, the strict inclusion criteria implemented had a twofold consequence. From one side, a conspicuous number of papers were discarded and those actually included presented a very high heterogeneity in terms of designs of the studies, participants’ numerosity and results presented. Direct consequences of these results on our work were the presentation of findings through a narrative synthesis and the inability to find a proper tool for the evaluation of the methodological quality of the included studies. Nevertheless, this allowed us to focus on the state of the art of interest, excluding new controllers not yet tested on stroke patients or clinical RCTs with no details on the technical aspects of the device or the control strategies. Hence, by focussing the review on the early stages of experiments with the implemented control strategies, we could provide a summary of the outcomes measures used at this stage.

Various study designs, heterogeneous outcome measures and high variability in patients’ stroke severity, methodologically affect the actual possibilities of developing protocols for a proper clinical- and patient-based selection of control strategies in exoskeletons for rehabilitation. Hence, our review mainly highlighted a lack of consensus in the selection of control strategies and outcomes on which beneficial results are expected in exoskeleton-based treatments and during daily assistance. These results may be related to a strong heterogeneity in the selection of the outcomes measures, and a yet limited clinical and patient-centred approach during the early development of exoskeletons. However, despite these limitations, our analysis showed awareness of the selection of the control strategy and robot characteristics for *assistive* or *therapeutic* purposes. Indeed, less compliant control strategies were found to be more frequently adopted within *assistive* contexts, whilst in *therapeutic* ones, where comfort, usability and compliance are required, force control strategies were preferred. These findings promote a promising clinical and patient-oriented approach for the design of wearable robotic devices. Moreover, they confirm the need for standardization in outcome measures used for the assessments after robot-aided gait rehabilitation. Shared consensus on the outcomes would promote the production of reliable evidence (meta-analyses) and be crucial for the identification of hidden patterns between the technical characteristics of the devices and the clinical results on the patients.

## Conclusions

As a key aspect for robotic-assisted rehabilitation, the selection of human–robot interaction, and specifically of a proper control strategy, is essential. Indeed, focussing on a human-centred and clinical based selection of the control strategy characteristics could allow for greater effectiveness and usability of both therapies and assistance in rehabilitation. Despite in the literature there is already a tendency toward a selection of more robust solutions for therapeutic applications and adaptive controllers for assistive ones, still, there is no consensus on the selection of controller type with respect to specific expected outcomes. For this reason, from the early steps of exoskeletons development, a translational approach, where both the technical requirements and the clinical ones should be considered.

## Data Availability

All data generated or analysed during this study are included in this published article and its supplementary information files.

## Abbreviations

AI: Artificial Intelligence
sEMG: Surface electromyography
EEG: Electroencephalography
PRISMA: Preferred Reporting Items for Systematic reviews and Meta-Analyses
PICO: Population, intervention, comparison, outcome
CHARMS: CHecklist for critical appraisal and data extraction for systematic reviews of prediction modelling studies
RCT: Randomized Controlled Trial
CT: Controlled Trial
NCT: Non-Controlled Trial
DoF: Degree of Freedom
GRF: Ground Reaction Force
RoM: Range of Motion
PID: Proportional-Integral-Derivative
TRL: Technology Readiness Level
CoP: Center of Pressure
MVC: Maximal Voluntary Contraction
10MWT: 10-Meters Walk Test
6MWT: 6-Minutes Walk Test
